# Cross-Cultural Adaptation and Psychometric Properties of the Chinese Version of the Knee Osteoarthritis Fears and Beliefs Questionnaire

**DOI:** 10.3390/healthcare12030310

**Published:** 2024-01-25

**Authors:** Shan Su, Clare Chung-Wah Yu, Gladys Lai-Ying Cheing, Raymond Chi-Keung Chung, Sharon Man-Ha Tsang, Lok-Lok Chan, Tracy Wing-Shan Tang, Winky Cheung, Qunn Jid Lee, Patrick Wai-Hang Kwong

**Affiliations:** 1 Department of Rehabilitation Sciences, The Hong Kong Polytechnic University, Kowloon, Hong Kong; susan2019.su@connect.polyu.hk (S.S.); gladys.cheing@polyu.edu.hk (G.L.-Y.C.); raymond.ck.chung@polyu.edu.hk (R.C.-K.C.); sharon.tsang@polyu.edu.hk (S.M.-H.T.); 22120912g@connect.polyu.hk (L.-L.C.); wai-hang.kwong@polyu.edu.hk (P.W.-H.K.); 2 Physiotherapy Department, Yan Chai Hospital, New Territories, Hong Kong; tws621@ha.org.hk (T.W.-S.T.); winky.cheung@ha.org.hk (W.C.); 3 Department of Orthopaedics and Traumatology, Yan Chai Hospital, New Territories, Hong Kong; leeqj@ha.org.hk

**Keywords:** knee, pain, fears, beliefs, validity, reliability

## Abstract

This study aimed to adapt and validate the Knee Osteoarthritis Fears and Beliefs Questionnaire (KOFBeQ) for Chinese patients, thereby advancing the understanding of fear-avoidance behaviors. Adopting a cross-sectional design, data were collected for 241 subjects (78.8% women; mean age 68.0 ± 7.8 years) across various healthcare settings in Hong Kong. Exploratory factor analysis resulted in an 11-item questionnaire with three distinct subscales, covering fears and beliefs related to physicians and disease (six items), daily living activities (three items), and sports and leisure activities (two items). The overall Cronbach’s α coefficient was 0.86, indicating strong internal consistency. The questionnaire exhibited favorable convergent validity. Confirmatory factor analyses confirmed a good model fit. Test–retest reliability analysis indicated a high intraclass correlation coefficient of 0.93 (95% confidence interval: 0.88, 0.96), and a Bland–Altman plot revealed a slight bias in two measurements (0.97 [0.19]) without a systematic trend. The adapted Chinese version of the KOFBeQ demonstrated robust psychometric properties in terms of validity and reliability, providing an effective tool for surveying Chinese patients with knee osteoarthritis. These findings offer valuable insights for clinicians and patients, aiding in informed decision-making and improved rehabilitation strategies.

## 1. Introduction

Knee osteoarthritis (OA) is a leading cause of disability in the older population, with global prevalence rates of up to 16% and over 30% in older individuals [1,2]. As China’s older demographic is projected to double from 168 million in 2010 to 402 million by 2040 [3], the population suffering from knee OA will be huge and growing in the coming years. Currently, there is no curable treatment, and exercise remains the primary recommendation for manage knee OA pain [4].

Knee OA pain is a type of chronic pain. Fear-avoidance beliefs exert influence on chronic pain and disability. Because beliefs can reshape human behaviors on the basis of reasonable information and thus influence human decisions, experiences involving pain may elicit fearful responses to other forms of exposure that are similar but not dangerous or painful [5]. Fears and beliefs have been reported to affect treatment adherence and prognosis of patients with back or neck pain [6,7]. Considering the influence of fear-avoidance beliefs on chronic pain, especially the potential influence of these fears and beliefs on the prognosis and treatment adherence of knee OA, evaluating patients’ fears and beliefs about knee OA is vital. The already available Chinese version of the Fear Avoidance Beliefs Questionnaire focuses on patients with low back pain [8], which is unlikely to extend the use of this scale in knee OA population. A validated tool for the Chinese population is currently unavailable, highlighting the need for a reliable instrument to facilitate effective disease management.

The Knee Osteoarthritis Fears and Beliefs Questionnaire (KOFBeQ) was developed to examine the fears and beliefs in patients with knee OA by Benhamou in 2013 [5]. It is an 11-item questionnaire with score ranges from 0 to 99 and four subscales, including fears and beliefs about daily living activities (three items), fears and beliefs about physicians (four items), fears and beliefs about the disease (two items), fears and beliefs about sports and leisure activities (two items) [5]. The higher scores indicate higher fears and beliefs. KOFBeQ was initially developed in France, with good validity and reliability. Fears and beliefs (KOFBeQ) were increased in patients with other comorbidities (e.g., obesity), affecting physical activity in patients with knee OA [9,10]. However, a validated Chinese version of this questionnaire is yet to be established. The present study aimed to translate and validate the KOFBeQ in a Chinese reading population. This work tried to expand the use of the questionnaire in patients with knee OA and facilitate application in assessing the clinical intervention. The internal consistency, construct validity, and test–retest reliability of the Chinese adaption of KOFBeQ were examined. It was hypothesized that the Chinese version of KOFBeQ would be a reliable and valid tool for patients with knee OA in the Chinese reading population.

## 2. Materials and Methods

### 2.1. Subjects

The present study recruited patients with (1) an age of more than 50 years; and (2) physician-diagnosed knee OA. These patients were recruited from local hospital outpatient physiotherapy departments, private physiotherapy clinics, and a community service center. All the subjects were required to be able to comprehend Standard Chinese. In addition, relevant sociodemographic and medical data of the subjects were collected. The present study excluded illiterate subjects with comorbidities, including a recent history of knee trauma, rheumatoid arthritis, or ligamentous injuries that affected their knee condition. This work was performed under the approval of the Human Subjects Ethics Subcommittee of Hong Kong Polytechnic University (HSEARS20220324004) and the Kowloon West Cluster Research Ethics Committee of the Hong Kong Hospital Authority [KW/EX-22-058(174-05)]. The procedures followed were in accordance with the ethical standards of the responsible institutional and national Committees on Human Experimentation, the principles of the Helsinki Declaration of 1975, as revised in 2000. All included subjects signed a consent form before data collection.

### 2.2. Sample Size Estimation

To validate the scale of the proposed questionnaire, an item-to-subject ratio of approximately 5 to 10 observations for each item is required and larger samples sizes are better [11]. Thus, for accurate results, a group of approximate 100 subjects was required to perform the exploratory factor analysis (EFA). Furthermore, another group of approximate 100 subjects was required to perform the confirmatory factor analysis (CFA). Therefore, a sample size of at fewest 200 subjects was required for the present study. For test–retest reliability, the previously reported intraclass correlation coefficient (ICC) was 0.81 and the width of the 95% confidence interval (CI) was 0.26 [5]; then, a group of at least 52 subjects was required to perform a reliability test [12].

### 2.3. Development of Chinese KOFBeQ

The development of cross-cultural adaptation was followed by the standard rules as recommended [13]. The original English version of the KOFBeQ was initially forward translated into Chinese by two independent translators. With the assistance of an observer, a synthesized version of translated questionnaires was produced after two translators’ consensus on resolving discrepancies. Then, two native speakers blinded to the original English version of the KOFBeQ were invited to back translate the preliminary Chinese version into English. An expert committee consisting of physicians, physiotherapists, and orthopaedic surgeons reviewed the original and back-translated English versions of the KOFBeQ and made necessary revisions to the Chinese version accordingly. The pre-final version of the translated questionnaire was produced after the expert panel reached a consensus on the discrepancies. Then, all written reports were submitted and appraised by developers. Because the Chinese version of the KOFBeQ did not exhibit any cultural or conceptual maladaptation, no item was changed or deleted.

### 2.4. Test Procedure

Eligible individuals who met the screening criteria were invited to respond to a set of the Chinese version of questionnaires, namely, (1) the KOFBeQ, (2) the Western Ontario and McMaster Universities OA index (WOMAC; a higher score indicates a worse condition) [14], (3) the Knee Injury and Osteoarthritis Outcome Score (KOOS; a lower score suggests a worse condition) [15], and (4) the International Physical Activity Questionnaire short form (IPAQ) [16]. Using the data collected with IPAQ, the volume of activity was calculated by weighting each type of activity by its energy requirements defined in METs (METs are multiples of the resting metabolic rate) and multiplying corresponding time to yield a score in MET-minutes per week (MET-min/week). For the levels of physical activity, patients were coded as low if total physical activity < 600 MET-min/week, moderate if >600 MET-min/week and <3000 MET-min/week, and high if ≥3000 MET-min/week. One week after, 59 randomly selected subjects were invited to participate in a second round of data collection over the phone to assess the test–retest reliability of the translated Chinese KOFBeQ.

### 2.5. Data Analysis

#### 2.5.1. Factor Analysis

Initially, EFA combined with principal component analysis (PCA) was performed to examine the construct validity of the Chinese KOFBeQ in a sample of 100 patients. To facilitate factor loading interpretation, varimax rotation combined with Kaiser normalization was performed. The factors generated through PCA were extracted if their eigenvalues were >1 [17]. An item was loaded onto a factor if its factor loading coefficient was >0.5. If an item could be loaded onto multiple factors, it was assigned to the factor with largest factor loading coefficients. Next, through the Amos program of SPSS, a separate sample of 141 patients was established to conduct CFA. Data were cleaned before pooling and inspection to ensure normality distribution. A CFA analysis model, which was built through maximum likelihood estimation, was designed to evaluate the fit of the data to the EFA-generated factor model. Goodness-of-fit indices (i.e., the goodness of fit index (GFI), the incremental fit index (IFI), and the comparative fit index (CFI)) were computed, and for the CFA model, a value of ≥0.9 indicated a perfect data fit, and a root mean square error of approximation (RMSEA) of <0.08 indicated acceptable data fit. The Chi-square divided by the degree of freedom ( $X^{2}/\mathrm{df}$ ) was also computed and a value of 3 or less was considered as acceptable.

#### 2.5.2. Internal Consistency

The internal consistency of the Chinese KOFBeQ was assessed using Cronbach’s α coefficient to examine the degree to which the separate items on the scale measured the same concept; in this context, the Cronbach’s α ranges of >0.70, 0.71–0.80, and >0.8 were regarded as acceptable, respectable, and excellent results, respectively [18]. The 95% CI of each Cronbach’s α value was assessed using the bootstrap technique with 1000 replications.

#### 2.5.3. Convergent Validity

Convergent validity was assessed by determining the Spearman correlation of the subscale scores and the global score of the Chinese KOFBeQ with other outcome measures (i.e., WOMAC, KOOS), and the differences of KOFBeQ scores across levels of physical activity analyzed using the non-parametric Kruskal–Wallis test.

#### 2.5.4. Test–Retest Reliability

To assess test–retest reliability on the basis of the ICC and a two-way random-effects model, 59 patients were randomly selected to undergo a second round of testing after one week [19]. An ICC of ≥0.75 indicated excellent reproducibility [20]. Test–retest reliability was also assessed using the Bland–Altman method, namely, plotting the difference between the two measurements for each subject against their mean. The limits of agreement (LoA) were calculated based on the mean of difference (MD) and the standard deviation (SD) ( 95%LoA=MD±1.96×SD ). The 95% CI for the limits is ±1.96×SE , where SE denotes as an approximation of standard error of limits ( $\mathrm{SE}=\sqrt{3{\mathrm{SD}}^{2}/N}$ ) [21]. If the values of difference between measurements violated the assumption of normality, homogeneity of variability, or proportional bias, the LoA would be derived from the transformed data by calculating the ratio of two measurements (T2/T1) for each subject [21]. The standard error of measurement (SEM) was estimated using the square root of the within-subject variance ( $\mathrm{SEM}=\mathrm{SDdiff}\times \sqrt{\left(1-r\right)}$ ), where “SD” denotes the SD of the observed test scores of a group of subjects, and r denotes the reliability of the coefficient for the measurement. The smallest detectable change at 90% CI (SDC90) was calculated by applying the following formula: $\mathrm{SDC}90=1.65\times \sqrt{2}\times \mathrm{SEM}$ [22]. The 1.65 in the SDC90 equation represents the z-score at the 90% confidence level.

All analyses were performed using SPSS (IBM SPSS Statistics for Windows, Version 26.0; IBM, Armonk, NY, USA).

## 3. Results

### 3.1. Descriptive Statistics

In total, 241 patients were recruited to participate in the present survey. All the subjects completed the Chinese KOFBeQ questionnaire and were included in subsequent analyses. Subjects’ demographics and characteristics are shown in Table 1. The mean (SD) value for age was 68.0 (7.8) years, and the mean disease duration was 6.9 (5.4) years. Women accounted for 78.8% of the study population. The IPAQ results indicated that 37.8% of the subjects engaged in high levels of physical activity, whereas 50.4% and 11.8% engaged in moderate and low levels, respectively. The median (interquartile range, IQR) time that they spent seated was 4 (2–5) hours/day.

### 3.2. Validity of Chinese KOFBeQ

#### 3.2.1. Data Completeness

No missing values were identified across the items and subscales (Table 2). Item 6 and item 10 simultaneously have ceiling and floor effects. The KOFBeQ subscales and global score did not exhibit a ceiling effect. However, a floor effect was observed for the sports subscale (19.1%).

#### 3.2.2. Factor Analysis

**Figure 1:**
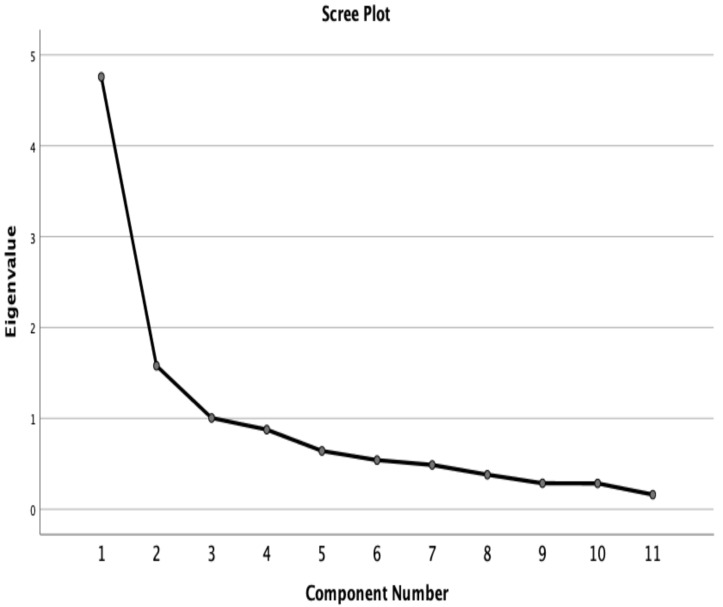
Exploratory factor analysis for the Knee Osteoarthritis Fears and Beliefs (n = 100).

**Figure 2:**
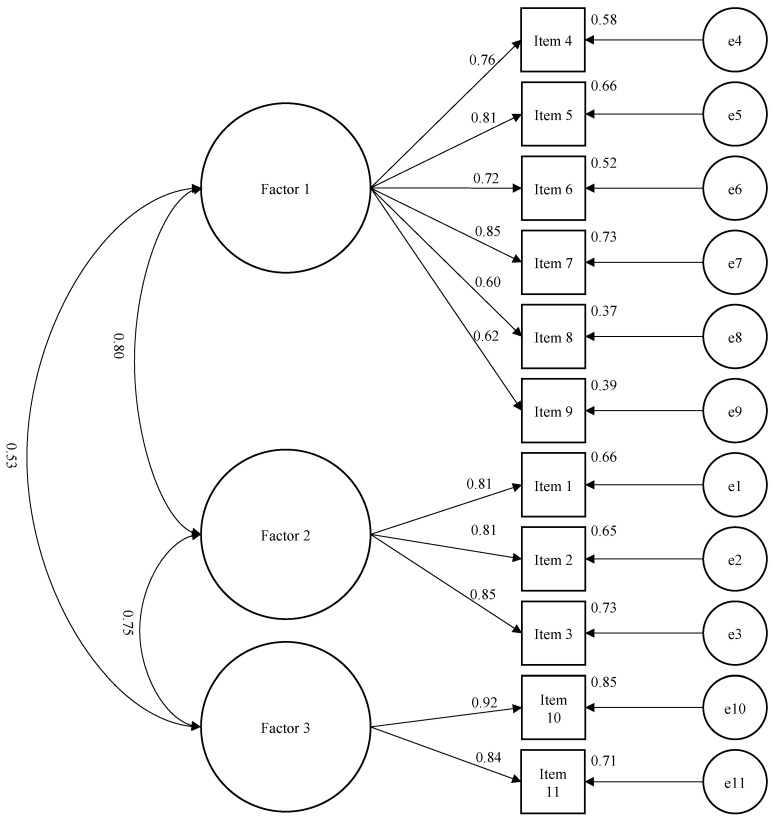
A confirmatory factor analysis using AMOS to test the feasibility of a model with factor 1 (physicians and disease), factor 2 (ADLs), and factor 3 (sports) in assessing fears and beliefs in patients with knee OA on eleven items. Residual values (e1–e11) represented variance not explained by the factors. Tests of goodness-of-fit suggested this model was a good fit for the observed data ( $X^{2}$ /df = 1.78, GFI = 0.91, IFI = 0.93, CFI = 0.93, RMSEA = 0.075). Standardized parameters were shown (n = 141).

Through EFA, three main factors with eigenvalues of 4.758, 1.579, and 1.005 were extracted, and these factors explained 66.75% of the total variance (Figure 1). The characterization of each factor was straightforward; specifically, factor 1 (six items) assessed fears and beliefs about physicians and disease, factor 2 (three items) assessed the ADLs, and factor 3 (two items) assessed sports (Table 3). Through CFA, the three-factor solution exhibited a good fit ( $X^{2}$ /df = 1.78, GFI = 0.91, IFI = 0.93, CFI = 0.93, RMSEA = 0.075), indicating that the three distinct factors could be used to assess different types of fears and beliefs about knee OA (Figure 2).

#### 3.2.3. Internal Consistency

The overall internal consistency was strong and acceptable given that a Cronbach’s α coefficient of 0.86 was obtained. The Cronbach’s α coefficients obtained when each item was deleted ranged from 0.84 to 0.85 (Table 4).

#### 3.2.4. Convergent Validity

KOFBeQ scores were revealed to be significantly correlated with WOMAC and KOOS scores. However, the obtained KOFBeQ scores did not significantly differ across the IPAQ levels of physical activity (i.e., low, moderate, high; Table 5).

#### 3.2.5. Test–Retest Reliability

**Figure 3:**
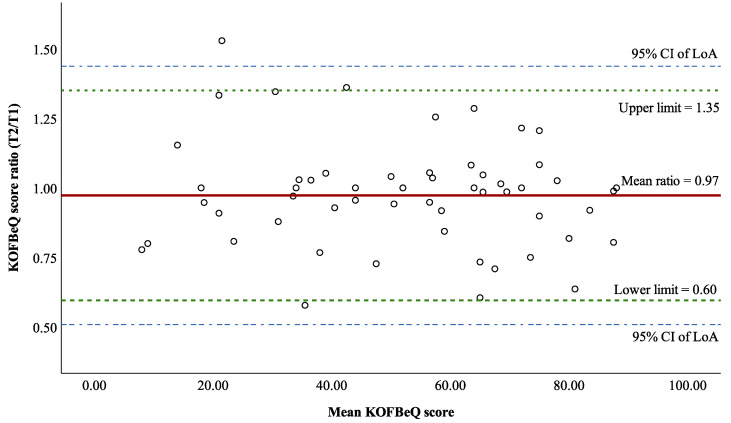
Bland–Altman plot of the Knee Osteoarthritis Fears and Beliefs Questionnaire global score.

Overall, 59 subjects recompleted the KOFBeQ questionnaire for the test–retest analysis, and the results indicated high test–retest reliability (Table 6), with an ICC of 0.93 (95% CI: 0.88, 0.96). The MD and SD of two measurements were −1.4±12.2 . For the SEM (3.2), the SDC90 was 7.4, which represented a smallest detectable change at 90% CI for KOFBeQ global score. Transformed data were used in the Bland–Altman analysis with 56 subjects (three outliers being excluded). The Bland–Altman analysis revealed a slight bias between the two assessments ( 0.97±0.19 ) without a systematic trend, and 95% of the values was determined to be randomly and uniformly distributed within LoA with 95% confidence (0.51, 1.44) (Figure 3).

## 4. Discussion

The present study validated a Chinese version of the KOFBeQ, which presented similar to the original version, exhibited excellent internal consistency, significant convergent validity, satisfactory construct validity, and high test–retest reliability. The findings of the present study suggested that the proposed Chinese version of the KOFBeQ was a reliable and valid outcome measurement tool for Chinese populations.

The main strength of the present study was its employment of PCA. The factorial structure of the Chinese KOFBeQ differed from that of the original KOFBeQ (four factors) in that three factors were identified after PCA and confirmed through CFA with a good fit. Subsequently, the items related to physicians and disease were in one factor with the highest eigenvalue, then ADLs and sports (Table 3). This finding may be attributed to cultural adaptation [23], namely the difference between the medical systems of Hong Kong and Western countries. In the initial study of the English version of the KOFBeQ, the participating patients were recruited from primary healthcare institutions [5]; compared with other institutions, primary healthcare institutions typically provide patients with continuing and preventive care [24]. For example, patients can receive health advice from doctors, physiotherapists, and dietitians for disease management and are encouraged to undertake more responsibility for managing their symptoms instead of relying on physicians [24]. In Hong Kong, the primary healthcare setting is currently underdeveloped, and patients mainly see doctors for acute episodes [25]. In addition, the present study mainly recruited its subjects from a local hospital, which is regarded as a secondary healthcare setting. OA patients who visit this hospital acquire most of their knowledge about knee OA from orthopedic specialists. Therefore, for the subjects of the present study, their understanding of the disease may be influenced by their physicians, and this feature could have influenced the results of the factor pertaining to the subjects’ fears and beliefs about physicians and disease. This possibility may explain why the factors identified in the Chinese KOFBeQ differed from those of the original KOFBeQ. Ensuring the validity of a set of items and concepts in the context of another language and culture is crucial; however, potential mismatches between readers of different languages can be difficult to address.

The Chinese KOFBeQ exhibited favorable psychometric properties. First of all, it presented a high internal consistency in terms of overall score and each item, indicating that all 11 items were essential for the scale [26]. The high ICC could be explained by the duration of knee OA. The history of knee OA in the studied population was approximately 7 years. Additionally, most of the patients underwent and became accustomed to multiple treatments and engaged in moderate to high levels of physical activity. These findings indicated that their conditions were stable, which could also contribute to the high test–retest reliability of the global score for the two measurements. Overall, the Chinese KOFBeQ exhibited favorable psychometric properties.

The Chinese KOFBeQ exhibited satisfactory convergent validity. In the present study, the Chinese versions of the WOMAC and KOOS were used to evaluate the convergent validity of the Chinese KOFBeQ because of their high validity and reliability [14,15] and some similar concepts as KOFBeQ. The results of the present study were similar to those of another study, which reported that the global score of the KOFBeQ was significantly correlated with knee pain and function [5]. In addition, the present study identified correlations among subscales. Fair to moderate correlations were identified between the Chinese KOFBeQ subscales (ADLs and sports) and the WOMAC and KOOS subscales. The physicians and disease subscale of the Chinese KOFBeQ exhibited a weak correlation with the WOMAC and KOOS subscales; however, this was expected because this subscale measured only the fears and beliefs with respect to physicians and disease, whereas the WOMAC and KOOS were designed to assess knee-related symptoms, function, and quality of life [27,28].

### Limitations

This study is not free of limitations. First of all, item 6 and item 10 simultaneously have ceiling and floor effects. The effects can be attributed to the sample being selected from two sources: (1) a hospital, where OA patients can visit the orthopedic specialists and have more severe symptoms and (2) community service centers, where OA patients have fewer opportunities to visit orthopedic specialists and more mild symptoms. Considering the high requirement for visiting the orthopedic specialists and having sports activity, these selected subjects are very likely to have extreme responses at two directions. Furthermore, the KOFBeQ exhibited a significant floor effect (19.1%) for the sports subscale (Table 2). This effect could also be attributed to the selected population. Specifically, our subjects were recruited from hospitals and community service centers, and they were typically individuals who played active roles in their treatment. Notably, nearly 90% of the subjects presented moderate to high levels of physical activity. The possible ways to address these issues are to ensure a diverse sample to reduce the risk of biased responses. Another limitation pertained to the use of a self-reported questionnaire to measure physical activity levels. Although fears and beliefs were hypothesized to affect physical activities, the KOFBeQ questionnaire was not associated with the IPAQ across the levels of physical activity (low, moderate, and high). This finding could be related to the characteristics of the subjects who are more likely to be physically active because of the active intervention and encouragement from their physicians. Also, the trend of older adults being employed is increasing in Hong Kong; a total of 139,300 adults aged ≥65 are engaged in employment, which is equivalent to a labor force participation rate of 11.7%. Thus, many of the subjects (aged > 50) may still have been working at the time of the current study. Another consideration is the recall bias associated with the IPAQ, which requires subjects to self-report their physical activities in the preceding 7 days [16]. A systematic review suggested that relative to objective measurements (e.g., accelerometer), the IPAQ tended to overestimate physical activity levels by 36–173% [29]. Therefore, many of the subjects in the present study could have remained physically active regardless of their fears and beliefs. To address this limitation, researchers should objectively measure physical activities to improve the ability to differentiate among levels of physical activity. Finally, the responsiveness of the Chinese KOFBeQ remains unclear. Thus, future studies should focus on the responsiveness of the questionnaire in the context of interventions (e.g., prognosis and adherence) and the relationship between the changes of responsiveness and clinical changes (i.e., psychological status and disability).

## 5. Conclusions

The introduction of the Knee Osteoarthritis Fears and Beliefs Questionnaire (KOFBeQ) for Chinese patients with knee OA serves as a valuable tool for clinicians and patients alike. By providing a comprehensive assessment across three distinct subscales—physicians and disease, ADLs, and sports—the Chinese KOFBeQ empowers clinicians to gain crucial insights into their patients’ fears and beliefs. With its strong internal consistency, validity, and high test–retest reliability, this questionnaire can effectively guide decision-making and treatment planning, enabling clinicians and patients to make more informed choices regarding knee OA management in the Chinese population.

## Figures and Tables

**Table 1:** Summary of characteristics.

	N	Mean (SD) or Median (IQR) or n (%)
**Sociodemographic characteristics**		
Age (years)	241	68.0 (7.8)
Female, n (%)	241	190 (78.8)
Height (cm)	241	156.8 (7.9)
Weight (kg)	240	63.3 (12.2)
BMI (kg/cm^2^)	240	25.8 (5.0)
**IPAQ, median (IQR)**		
Total MET-min/week	238	2056.5 (1053.0–4678.5)
Walking MET-min/week	238	1368.0 (693–2772)
Moderate MET-min/week	238	240.0 (0–740)
Vigorous MET-min/week	238	0 (0–0)
Sitting hours/day	238	4 (2–5)
**Levels of physical activity, n (%)**		
Low		28 (11.8)
Moderate	238	120 (50.4)
High		90 (37.8)
**Medical status**		
Duration of knee OA (years)	240	6.9 (5.4)
**Side of knee OA, n (%)**		
Left		43 (17.8)
Right	240	34 (14.1)
Both		162 (67.2)
**Functional status**		
WOMAC pain	240	8.0 (4.1)
WOMAC stiffness	240	3.1 (1.7)
WOMAC function	240	23.8 (13.3)
WOMAC global score	240	34.9 (17.9)
KOOS symptom	240	57.3 (20.2)
KOOS pain	240	61.8 (19.8)
KOOS ADLs	240	67.3 (20.4)
KOOS Sports&Recreation	240	35.5 (27.7)
KOOS QoL	240	44.4 (23.9)

Note: For the levels of physical activity, patients were coded as low if total physical activity < 600 MET-min/week, moderate if >600 MET-min/week and <3000 MET-min/week, high if ≥3000 MET-min/week; SD: standard deviation; IQR: interquartile range; BMI: body mass index; IPAQ: the International Physical Activity Questionnaire; WOMAC:Western Ontario and McMaster Universities Osteoarthritis Index; KOOS: Knee Injury and Osteoarthritis Outcome Score; ADLs: daily living activities; QoL: quality of life.

**Table 2:** Score distributions of the items and subscales of the Knee Osteoarthritis Fears and Beliefs Questionnaire (n = 241).

	Missing Values (%)	Mean (SD)	Median	Range	Floor Effect (%)	Ceiling Effect (%)
**Items**						
Item 1	0	-	4	0–9	13.3	19.5
Item 2	0	-	3	0–9	22.8	9.5
Item 3	0	-	5	0–9	7.9	22.8
Item 4	0	-	4	0–9	13.3	15.8
Item 5	0	-	5	0–9	7.5	17.4
Item 6	0	-	5	0–9	15.8	23.2
Item 7	0	-	4	0–9	17.0	13.3
Item 8	0	-	4	0–9	22.0	12.0
Item 9	0	-	4	0–9	15.8	12.0
Item 10	0	-	4	0–9	22.4	25.3
Item 11	0	-	3	0–9	27.0	12.4
**Subscales**						
Physicians & Disease	0	13.1 (7.8)	13	0–54	2.1	1.7
ADLs	0	26.9 (12.6)	26	0–27	6.2	6.6
Sports	0	8.1 (6.2)	8	0–18	19.1	10.8
Global score	0	48.1 (21.7)	46	0–99	0.4	1.2

Note: criteria for ceiling effect or floor effect ≥ 15%; ADLs: daily living activities.

**Table 3:** Factor loadings from the final solution of factor analysis for the Knee Osteoarthritis Fears and Beliefs Questionnaire (n = 141).

Variable	Factor 1	Factor 2	Factor 3
**Factor 1 (Physicians and Disease)**			
Item 7	0.744		
Item 5	0.733		
Item 4	0.703		
Item 6	0.636		
Item 8	0.606		
Item 9	0.579		
**Factor 2 (ADLs)**			
Item 1		0.899	
Item 3		0.810	
Item 2		0.682	0.446
**Factor 3 (Sports)**			
Item 10			0.869
Item 11			0.856

Note: ADLs: daily living activities.

**Table 4:** Internal consistency of the Knee Osteoarthritis Fears and Beliefs Questionnaire (Overall Cronbach’s α = 0.857) (n = 241).

	Scale Mean If Item Deleted	Scale Variance If Item Deleted	Corrected Item-Total Correlation	Squared Multiple Correlation	Cronbach’s α If Item Deleted
Item 1	46.4398	398.281	0.529	0.556	0.847
Item 2	45.2697	392.373	0.604	0.487	0.841
Item 3	47.0041	388.212	0.634	0.594	0.839
Item 4	46.4315	402.396	0.517	0.418	0.848
Item 5	47.0249	406.308	0.521	0.453	0.847
Item 6	46.8423	396.392	0.503	0.367	0.849
Item 7	46.0041	407.654	0.460	0.353	0.852
Item 8	45.7095	400.340	0.508	0.477	0.848
Item 9	46.0000	390.558	0.611	0.552	0.841
Item 10	46.3776	379.369	0.601	0.634	0.841
Item 11	45.4440	393.673	0.537	0.592	0.846

Note: Cronbach’s α > 0.80 was regarded as excellent result.

**Table 5:** Convergent validity of the Knee Osteoarthritis Fears and Beliefs Questionnaire.

		KOFBeQ		
	**Physicians and Disease**	**ADLs**	**Sports**	**Global Score**
**Correlation of the subscales and global scores of the KOFBeQ with the other knee-related scores**				
WOMAC pain	0.223 †	0.465 †	0.353 †	0.408 †
WOMAC stiffness	0.188 †	0.385 †	0.328 †	0.342 †
WOMAC physical function	0.223 †	0.439 †	0.325 †	0.394 †
WOMAC total score	0.230 †	0.465 †	0.343 †	0.412 †
KOOS symptom	−0.183 †	−0.383 †	−0.317 †	−0.336 †
KOOS pain	−0.186 †	−0.496 †	−0.339 †	−0.394 †
KOOS ADLs	−0.219 †	−0.482 †	−0.350 †	−0.416 †
KOOS Sports & Recreation	−0.179 †	−0.449 †	−0.327 †	−0.367 †
KOOS QoL	−0.147 *	−0.419 †	−0.309 †	−0.336 †
**Comparing the scores of KOFBeQ across levels of physical activity**				
Low	27.46 (12.34)	15.86 (7.99)	9.96 (6.77)	53.29 (21.51)
Moderate	27.69 (12.53)	13.20 (7.39)	8.44 (6.30)	49.33 (21.65)
High	26.40 (12.37)	12.46 (8.21)	7.27 (5.81)	46.12 (21.24)
*p*-value	0.696	0.127	0.122	0.246

Note: * *p* < 0.05; † *p* < 0.01; For the levels of physical activity, patients were coded as low if total physicalactivity <600 MET-min/week, moderate if >600 MET-min/week and <3000 MET-min/week, high if ≥3000 MET-min/week; KOFBeQ: Knee Osteoarthritis Fears and Beliefs Questionnaire; WOMAC: Western Ontario and McMaster Universities Osteoarthritis Index; KOOS: Knee Injury and Osteoarthritis Outcome Score; ADLs: daily living activities; QoL: quality of life.

**Table 6:** Test–retest reliability statistics of the Knee Osteoarthritis Fears and Beliefs Questionnaire.

	N	Mean at Baseline (SD)	Mean at 2nd Time (SD)	Mean Difference (SD)	ICC_2,1_ (95%CI)	SEM	SDC90
Global score	59	51.5 (24.8)	50.0 (21.4)	−1.4 (12.2)	0.93 (0.88, 0.96)	3.2	7.4

Note: ${\mathrm{ICC}}_{2,1}$ is an intraclass correlation coefficient model (2,1); $\mathrm{SEM}=\mathrm{SDdiff}\times \sqrt{\left(1-r\right)}$ ; $\mathrm{SDC}90=1.65\times \sqrt{2}\times \mathrm{SEM}$ ; ICC: intraclass correlation coefficient; SEM: standard error of measurement; SDC90: smallest detectable change at 90% confidence interval.

## Data Availability

Data are contained within the article.

## Abbreviations

The following abbreviations are used in this manuscript:

OA	Osteoarthritis
KOFBeQ	The Knee Osteoarthritis Fears and Beliefs Questionnaire
ADLs	Daily Living Activities
EFA	Exploratory Factor Analysis
CFA	Confirmatory Factor Analysis
CI	Confidence Interval
WOMAC	the Western Ontario and McMaster Universities OA index
KOOS	the Knee Injury and Osteoarthritis Outcome Score
IPAQ	the International Physical Activity Questionnaire short form
METs	multiples of the resting metabolic rate
PCA	principal component analysis
GFI	Goodness of Fit Index
IFI	Incremental Fit Index
CFI	the Comparative Fit Index
RMSEA	Root Mean Square Error of Approximation
$X^{2}$	Chi-square
df	degree of freedom
ICC	The Intraclass Correlation Coefficient
LoA	The limits of agreement
MD	The Mean of Difference
SD	Standard Deviation
SE	Standard Error
SEM	Standard Error of Measurement
SDC90	The Smallest Detectable Change at 90% confidence interval
IQR	interquartile range
